# Supplementary material for: Reliability of the thumb localizing test and its validity against quantitative measures with a robotic device in patients with hemiparetic stroke
Source: PLoS One. 2020 Jul 24;15(7):e0236437. doi: 10.1371/journal.pone.0236437 (PMC7380647; doi:10.1371/journal.pone.0236437)
Supplement: Attachment — Submitted filename: Response_to_Reviewers.docx [file pone.0236437.s003.docx]

Response to Reviewers

We are grateful for the helpful comments from the reviewers. We have provided point-by-point responses to all comments below.

*Reviewer #1: Dear study authors*

*Thank you for the opportunity to review your manuscript. You have undertaken a study to determine the inter-rater validity of the thumb localising test in a chronic stroke population, and to examine its correlation with the KINARM exoskeleton device.*

*Firstly, thank you for the figures. They are very useful in these types of studies. Your study is nicely summarised and clear, however I feel you are currently missing a few important details that would assist readers. I have provided some comments below for your consideration. I hope these are helpful.*

*Major comments.*

*Introduction / References: Your background studies and references and all starting to get quite old. There is newer work in this area. Can you please incorporate discussion of more recent findings throughout the manuscript, and further discuss the implications for your methods and results. How does this work fit into other studies in both stroke and proprioception?*

-response:

We appreciate this suggestion. We have included more recent studies in the Introduction.

page 3, line 47

In stroke patients, proprioceptive deficits are quite common [7, 8] and known to be partly related to motor recovery [9, 10]. Nevertheless, the details including its longitudinal process of recovery or effective treatment strategies are yet to be revealed, and it can be assumed that this is one of the reasons that robust assessments of proprioception have been lacking. Therefore, establishing proprioceptive assessment tools would promote understanding of proprioceptive recovery after stroke and bring new perspectives on rehabilitative approach.

page 5, line 81

To confirm the robustness of the TLT as a clinical measure of proprioception, the present study aimed (1) to examine the inter-rater reliability of the TLT, and (2) to validate the TLT by examining correlation with the quantitative measures derived from the exoskeleton robotic device.

References

8. Kessner SS, Schlemm E, Cheng B, Bingel U, Fiehler J, Gerloff C, et al. Somatosensory deficits after ischemic stroke. Stroke. 2019;50(5):1116-23. Epub 2019/04/05. doi: 10.1161/strokeaha.118.023750. PubMed PMID: 30943883.

10. Winters C, van Wegen EE, Daffertshofer A, Kwakkel G. Generalizability of the proportional recovery model for the upper extremity after an ischemic stroke. Neurorehabilitation and neural repair. 2015;29(7):614-22. Epub 2014/12/17. doi: 10.1177/1545968314562115. PubMed PMID: 25505223.

*Methods: As you are undertaking an inter-rater validity study, please provide details on the raters, including their training, experience, and professional background). Was the person helping the participant a different person to the assessors, and if so please also add details on who they are.*

-response:

Thank you for the instruction. We added detailed information about the two raters and also about the persons who served as examiners.

page 5, line 114

Before each limb position was fixed, the examiner moved the limb multi-directionally to prevent the subject from guessing the limb position based on the starting position.

page 6, line 133

To score the TLT, all performances were recorded on videotape and were independently rated afterwards by two physiatrists; EO with 6 years of clinical experience and YO, with 18 years of clinical experience. Three physiatrists, EO, YO, KY, with 5 years of clinical experience, and an occupational therapist, AN, with 12 years of clinical experience, served as examiners.

*Please explain the Fugl-Meyer and the Ashworth tests in more detail, including how you undertook them, the scoring (max score and how scored) and interpretation of the scores (i.e. a high Fugl-Meyer score is?). Also, please state which side you are reporting the Fugle-Meyer for (ie. More affected, less affected).*

-response:

Thank you for the suggestion. We have explained Fugl-Meyer Assessment and Modified Ashworth scale, including the interpretation in the Results part, and have modified Table 1 as follows.

page 5, line 103

Clinical Assessments

To evaluate the participants’ upper limb impairments, we assessed the affected arm using the Fugl-Meyer Assessment, the part A score of the upper limb section to quantify motor function (0 (worst) - 36 (best))[21], and the Modified Ashworth Scale for elbow flexors to grade spasticity (0(no spasticity), 1, 1+, 2, 3, 4(rigid))[22].

page 11, line 253

Participants with a wide range of motor impairments were included. Spasticity of the participants ranged from mild to moderate.

page 11, line 260: Table 1

Part A score of the Fugl-Meyer Assessment of Upper Extremity on the affected side, median (range)

Modified Ashworth scale for elbow flexors in the affected arm, n in [0, 1, 1+, 2, 3, 4]

References

21. Fugl-Meyer AR, Jaasko L, Leyman I, Olsson S, Steglind S. The post-stroke hemiplegic patient. 1. a method for evaluation of physical performance. Scandinavian journal of rehabilitation medicine. 1975;7(1):13-31. Epub 1975/01/01. PubMed PMID: 1135616.

22. Bohannon RW, Smith MB. Interrater reliability of a modified Ashworth scale of muscle spasticity. Physical therapy. 1987;67(2):206-7. Epub 1987/02/01. doi: 10.1093/ptj/67.2.206. PubMed PMID: 3809245.

*What are the time intervals between testing, were they all done on the same day, different days etc. how did you manage fatigue in these participants?*

-response:

Thank you for the question. We have added descriptions regarding testing time and management of participants’ fatigue during robotic assessment.

page 8, line 173

It took approximately 10-15 minutes per session, and all sessions were usually performed consecutively on the same day. Examiners routinely advised participants to take enough time and rest between the testing sessions to fully concentrate on the task, and most participants needed no rest or only a few minutes.

*Results: Please put the results for each variable in a table in addition to the figures. Especially for the TLT results which do not appear to be reported at present.*

-response:

Thank you for the suggestion. In addition to the ultimate scores of the TLT presented in Table 1, we reported all the scores of the TLT as Table 2. We have also added the values of robotic measures in Table 3.

page 11, line 257

The overall weighted kappa of the TLT scores between the two raters was 0.84 (*P* < 0.001), indicating almost perfect agreement (Table 2).

page 12, line 262: Table 2

**Table 2. Scores of the thumb localizing test and quadratically weighted kappa.**

|  | Scores of rater A, n in [0, 1, 2, 3] | Scores of rater B, n in [0, 1, 2, 3] | Quadratically weighted kappa | *P* values |
| --- | --- | --- | --- | --- |
| Position 1 | [15,17,1,7] | [15,15,5,5] | 0.86 | < 0.001 |
| Position 2 | [15,12,5,8] | [17,7,11,5] | 0.86 | < 0.001 |
| Position 3 | [20,8,9,3] | [17,7,11,5] | 0.81 | < 0.001 |
| Total | [50,37,15,18] | [49,29,27,15] | 0.84 | < 0.001 |

Position 1, the forearm in the neutral position, with the elbow at 90° of flexion, and the shoulder at 0° of flexion; Position 2, the forearm in the neutral position, with the elbow at 90° of flexion, and the shoulder flexed so that the thumb is at the same level as the mouth; Position 3, the forearm in the neutral position, with the elbow at 90° of flexion, and the shoulder internally rotated so that the thumb is over the midline of the trunk.

page 13, line 287

As shown in Table 3, in the correlational analyses between the TLT and the robotic measures, *Area* correlated most strongly with the TLT with vision occluded condition.

page 13, line 294: Table 3

**Table 3. Values of the robotic measures with vision occluded and correlations with thumb localizing test.**

| Measures | Median value  (range) | Spearman’s rho  (95% confidence intervals) | *P* values |
| --- | --- | --- | --- |
| *Variability* | 5.76  (2.34-18.78) | 0.40  (0.10 - 0.63) | 0.011 |
| *Area* | 0.56  (0.01-2.90) | -0.71  (-0.84 - -0.51) | < 0.001 |
| *Shift* | 6.35  (1.81-18.11) | 0.27  (-0.05 - 0.54) | 0.093 |

*Discussion: There are some terms in your discussion that have not been introduced earlier. Please give a short description of what you mean by these terms (i.e. kinaesthetic sensation, spatial cognition.*

-response:

Thank you for pointing out the specific terms to be described. We have added the corresponding sentences.

page 16, line 344

… and it also indicates that the TLT may reflect at least a part of “kinesthetic sensation” , which is generated from muscle spindles in response to limb movement and changes in position[31].

page 17, line 367

… and thus might have various confounding factors such as disturbance in spatial cognition due to cognitive impairments, including visuospatial neglect[29] or deficits in generalized attention.

Reference

29. Semrau JA, Herter TM, Scott SH, Dukelow SP. Vision of the upper limb fails to compensate for kinesthetic impairments in subacute stroke. Cortex; a journal devoted to the study of the nervous system and behavior. 2018;109:245-59. Epub 2018/11/06. doi: 10.1016/j.cortex.2018.09.022. PubMed PMID: 30391879.

*How do you think the changes that may have occurred in the brain after the stroke has influenced your findings?*

-response:

Thank you for your thought-provoking question. The degree of proprioceptive deficits represented by the TLT might be related to the lesions which are known to have importance in passive kinesthesic sensation or sensorimotor integration. We have therefore added to the limitation part as the present study did not include information regarding brain lesions in the analysis.

page 17, line 385

Finally, another study would be required to reveal the relationship between the proprioceptive deficits represented by the TLT and brain lesions that may affect passive kinesthesic sensation or sensorimotor integration, as indicated in recent studies[8, 29].

References

8. Kessner SS, Schlemm E, Cheng B, Bingel U, Fiehler J, Gerloff C, et al. Somatosensory deficits after ischemic stroke. Stroke. 2019;50(5):1116-23. Epub 2019/04/05. doi: 10.1161/strokeaha.118.023750. PubMed PMID: 30943883.

29. Semrau JA, Herter TM, Scott SH, Dukelow SP. Vision of the upper limb fails to compensate for kinesthetic impairments in subacute stroke. Cortex; a journal devoted to the study of the nervous system and behavior. 2018;109:245-59. Epub 2018/11/06. doi: 10.1016/j.cortex.2018.09.022. PubMed PMID: 30391879.

*Limitations: You could expand on your limitations section, particularly;*

*You have briefly mentioned it, but you seen to have a fairly young and very high functioning cohort. This does not seem overly typical of a normal stroke population. Similarly, do you think your test would be as accurate in a less high functioning population? Please expand on this limitation.*

*Are you likely to see these changes with age, or are they purely due to the stroke?*

-response:

Thank you for pointing out in detail the generalization of the present study. As you can see, the participants are relatively young and high functioning among the whole stroke population. We have also added the sentence regarding the generalizability of our findings to other pathological conditions. We expanded on the limitation part as follows, adding that point.

page 17, line 381

Second, the generalizability of our findings may be somewhat limited as the present study investigated chronic patients living independently, who were relatively young, and high functioning as compared to the general stroke population. In addition, further investigation will be needed to generalize our findings to individuals with other pathological conditions.

*You have mentioned in the introduction the TLT is only an ordinal test, however what does this for interpretation of your results.*

-response:

Thank you for the question. By means of confirming psychometric properties, the present study could intensify the clinical usefulness of the TLT, an ordinal scale, although ordinal scales generally have disadvantages as quantification tools compared with continuous scales. We mentioned that point as follows.

page 3, line 59

… and thus disadvantageous as a quantification tool in detecting subtle deficits or changes over time compared with continuous scales.

page 18, line 397

In spite of its ordinal nature of the scale, which tends to be a disadvantage when compared with continuous scales, the TLT has been proved to be essential as an easily performed and robust tool to detect proprioceptive deficits in clinical scenes.

*Minor comments:*

*Table 1: You may wish to relabel “types of disease”, as Stroke type, or type of stroke.*

-response:

Thank you for the suggestion. We relabeled as such (page 11, line 260: Table 1).

*Line 330 – typo – “grades” rather than grads.*

-response:

It is definitely right. We corrected the typo (page 17, line 374).

*309 – do you mean the study did “not” show significant correlations?*

-response:

Thank you for pointing out. We corrected the sentence (page 16, line 352).

*Reviewer #2: This is a well-written manuscript examining the inter-rater reliability of the Thumb Localizer Task in 40 participants with chronic stroke. Additionally, the authors make comparisons of the TLT results with those obtained from a well validated robotic measure of proprioception called position matching performed in the KINARM robot. The methodologic approach appears quite reasonable. My main suggestions with the paper are fairly minor.*

*The authors might consider making the point that the TLT is only a 4 point scale, and although it would theoretically be possible to get low reliability when doing such an experiment, not obtaining high inter-rater reliability from two trained observers scoring the same video of a patient would present quite a challenge.*

-response:

Thank you for your valuable comment. We actually obtained almost perfect inter-rate reliability in the present study (weighted kappa 0.84, *P*<0.001) according to the reference (Landis and Koch, Biometrics 1977). We changed the text as follows in order to emphasize that.

page 15, line 319

… this study revealed that the TLT had almost perfect inter-rater reliability in the standardized way.

*The authors should also take a little more time to explain why they used the “vision restored” conditions.*

-response:

Thank you for your advice. We have added the reason why the two vision conditions were assessed.

page 5, line 116

The participants performed the procedure twice, with vision occluded condition (with a blindfold) and with vision restored condition (without a blindfold). This was to analyze the effect of vision, as the importance of visual information in compensating proprioceptive deficit is clinically well known [4, 23].

References

23. Newport R, Hindle JV, Jackson SR. Links between vision and somatosensation. Vision can improve the felt position of the unseen hand. Curr Biol. 2001;11(12):975-80. Epub 2001/07/13. doi: 10.1016/s0960-9822(01)00266-4. PubMed PMID: 11448775.

*“though it is a four-point scale and thus unable to quantify the deficits.” Although I agree with the authors a 4 point ordinal scale isn’t exactly the most desirable tool to “quantify” something. However the definition of the word “quantify” basically means you are trying to measure something and the TLT does this. I would suggest rewording the sentence accordingly.*

-response:

Thank you for your suggestion. We reworded the sentence so that the difference between ordinal and continuous scales would be clearer.

page 3, line 59

… and thus disadvantageous as a quantification tool in detecting subtle deficits or changes over time compared with continuous scales.

*Figures 1, 2 – The image on the PDF file I received was quite pixelated. The authors should consider addressing this prior to publication (I have seen this happen with some of my own manuscripts when the file gets converted by the publisher’s software, sometimes changing the file type can be helpful).*

-response:

Thank you for the comment. We made the figures clearer. In addition, we changed the layout of Fig 3 to make it easier to look.

*Discussion*

*Figures 4, 5 - It would have been nice here to also see Variability and Shift plotted in a similar way against TLT. Consider turning these into 3 panel figures to display all the data.*

-response:

Thank you for the suggestion. We have modified the figures and corresponding parts in the Results and Discussion.

page 13, line 279

**Fig 4. Scatterplots of robotic measures with vision occluded and with vision restored in each grade of the thumb localizing test (TLT).** Scatterplots of (A)*Variability*, (B)*Area*, and (C)*Shift* are shown. The dots under the dotted line (y=x) indicate that the value is smaller with vision occluded than with vision restored, and the dots above the dotted line (y=x) indicate that the value is larger with vision occluded than with vision restored. The value of Area < 1 (gray shaded area) implies contraction and > 1 implies expansion of the workspace.

page 14, line 297

On performing the Kruskal-Wallis rank test, the values of *Variability* (P =0.049) and *Area* (P < 0.001) were found to be significantly different across the TLT rating groups, but the values of *Shift* were not (P=0.248). Multiple comparisons using Dunn’s post-hoc test revealed that the values for *Variability* were significantly different between the TLT scores of 0 and 2 (P=0.039), 0 and 3 (P<0.001), and 1 and 3 (P=0.017). As for the values for *Area*, significant differences were seen between the TLT scores of 0 and 1 (P = 0.027), 0 and 2 (P < 0.001), 0 and 3 (P < 0.001), and 1 and 3 (P = 0.015) (Fig 5).

page 14, line 306

**Fig 5. Robotic measures with vision occluded of the arm position matching task in each grade of the thumb localizing test (TLT).** Boxplots of (A)*Variability*, (B)*Area*, and (C)*Shift* are shown. Error bars indicate standard deviations and the lines within the box indicate median values. ** P < 0.01, * P < 0.05 with the Dunn’s test for multiple comparisons.

page 17, line 371

In the comparisons between the grades of TLT, *Variability* and *Area* were not statistically different and substantially overlapped between grade 1 and 2, and between 2 and 3 of the TLT score.

*Page 14 – “significantly deteriorated” implies that the vision restored condition was conducted first, then the vision occluded. I suspect the authors meant “was significantly worse” or similar.*

-response:

Thank you for your suggestion. As stated in the Methods part, we performed robotic assessments first with a shield above the arms so that vision was occluded, and then repeated the task with vision restored. Thus, we modified the expression as you suggested.

page 15, line 324

Similarly, all three robotic measures were significantly worse in the vision occluded condition, compared to the vision restored condition.

*The authors may want to consider reviewing/discussing/contrasting a larger study investigating the return of vision (Herter et al. Vision does not always help stroke survivors compensate for impaired limb position sense. J Neuroeng Rehabil 2019.) as it uses the same task and many subjects fail to improve with vision restored.*

-response:

We are grateful for your introduction of the helpful study. We added some of the recent studies as references.

page 15, line 327

These findings indicate that clinical measures and robotic assessments have the same characteristics. Although some studies report that vision does not always compensate for kinesthetic impairments in stroke [29, 30], the subjects in these studies were in the relatively early phases (average of less than 2 weeks post stroke). On the contrary, the present study was performed in the chronic phase, in which visuospatial deficits or fatigue were more likely to diminish compared to early or subacute phases post stroke, and thus visual compensation was considered to be more effective than the above-mentioned studies.

page 17, line 367

… and thus might have various confounding factors such as disturbance in spatial cognition due to cognitive impairments, including visuospatial neglect[29] or deficits in generalized attention.

References

29. Semrau JA, Herter TM, Scott SH, Dukelow SP. Vision of the upper limb fails to compensate for kinesthetic impairments in subacute stroke. Cortex; a journal devoted to the study of the nervous system and behavior. 2018;109:245-59. Epub 2018/11/06. doi: 10.1016/j.cortex.2018.09.022. PubMed PMID: 30391879.

30. Herter TM, Scott SH, Dukelow SP. Vision does not always help stroke survivors compensate for impaired limb position sense. J Neuroeng Rehabil. 2019;16(1):129. Epub 2019/11/02. doi: 10.1186/s12984-019-0596-7. PubMed PMID: 31666135; PubMed Central PMCID: PMCPMC6822422.
