# Supplementary material for: Reliability of the thumb localizing test and its validity against quantitative measures with a robotic device in patients with hemiparetic stroke
Source: PLoS One. 2020 Jul 24;15(7):e0236437. doi: 10.1371/journal.pone.0236437 (PMC7380647; doi:10.1371/journal.pone.0236437)
Supplement: Attachment — Submitted filename: Response_to_Reviewers.docx [file pone.0236437.s004.docx]

**Response to the reviewer**

We are grateful for the helpful comments from the reviewer. We have provided point-by-point responses to all comments below.

*Reviewer #2: Thanks to the authors for responding to my previous review. The paper appears much improved. I did catch a few minor details that they authors should likely deal with.*

*Line 50 – actually the longitudinal process following stroke recovery has been discussed in a few papers, one that uses the same device the authors are using in the present manuscript (see Semrau et al. 2015, Stroke “Examining Differences in Patterns of Sensory and Motor Recovery After Stroke With Robotics”. There are also a few papers that use clinical scales to look at recovery of proprioception and a reasonably sized recent one published in Neurorehabilitation and Neural Repair (“Zandvliet et al. 2020 34(5):403-426 “Is Recovery of Somatosensory Impairment Condition for Upper-limb motor recovery after stroke?”). So the authors may want to soften the existing comment and reference studies that have looked at it. I completely agree there is room for further, more detailed studies here, but they authors should not ignore the efforts of others that have already looked at this issue.*

-response:

We appreciate this suggestion. We modified the sentences and added the references.

page 3, line 48

As its longitudinal process of recovery has been partly revealed recently [8, 11, 12] and novel rehabilitative approaches are sought more than ever in clinical practice, indispensability for robust assessment tools of proprioception is increasing to accurately evaluate its improvement with progress of time or by any intervention.

References

8.	Kessner SS, Schlemm E, Cheng B, Bingel U, Fiehler J, Gerloff C, et al. Somatosensory Deficits After Ischemic Stroke. Stroke. 2019;50(5):1116-23. Epub 2019/04/05. doi: 10.1161/strokeaha.118.023750. PubMed PMID: 30943883.

11.	Semrau JA, Herter TM, Scott SH, Dukelow SP. Examining Differences in Patterns of Sensory and Motor Recovery After Stroke With Robotics. Stroke. 2015;46(12):3459-69. Epub 2015/11/07. doi: 10.1161/strokeaha.115.010750. PubMed PMID: 26542695.

12.	Zandvliet SB, Kwakkel G, Nijland RHM, van Wegen EEH, Meskers CGM. Is Recovery of Somatosensory Impairment Conditional for Upper-Limb Motor Recovery Early After Stroke? Neurorehabilitation and neural repair. 2020;34(5):403-16. Epub 2020/05/12. doi: 10.1177/1545968320907075. PubMed PMID: 32391744; PubMed Central PMCID: PMCPMC7222963.

*Line 106 – Fugl-Meyer. The upper score should be 66 here (I think the 36 is a typo).*

-response:

Thank you for your comment. The best score of upper limb section in total would be 66 as you mentioned. Herein, we provided only the part A score with a maximum score of 36 to focus on the general and proximal upper limb function.

*Line 314 – I suspect you mean “quantitative” rather than “qualitative” measure as it is written.*

-response:

Thank you for pointing this out. We changed this to “quantitative” (page 15, line 313).
